# Ethnic differences in beta cell function and pancreatic fat in Black African and White European men across a spectrum of glucose tolerance

**DOI:** 10.1007/s00125-025-06514-3

**Published:** 2025-08-06

**Authors:** Gráinne Whelehan, Olah Hakim, Meera Ladwa, Oluwatoyosi Bello, Danielle H. Bodicoat, A. Margot Umpleby, Stephanie A. Amiel, Louise M. Goff

**Affiliations:** 1 Diabetes Research Centre, University of Leicester, Leicester, UK; 2 NIHR Leicester Biomedical Research Centre, Leicester, UK; 3 School of Life and Health Sciences, University of Roehampton, London, UK; 4 Department of Diabetes, School of Life Course Science, Faculty of Life Sciences & Medicine, King’s College London, London, UK; 5 Independent Researcher, Leicester, UK; 6 Faculty of Health and Medical Sciences, University of Surrey, Guildford, UK

**Keywords:** Acute insulin response, Beta cell function, Ethnic differences, Insulin secretion

## Abstract

**Aims/hypothesis:**

People of Black African (BA) ancestry are disproportionately affected by type 2 diabetes when compared with people of White European (WE) descent, despite lower levels of ectopic fat. Impaired beta cell function is a key pathophysiological feature of type 2 diabetes. It remains to be determined whether an associative relationship exists between intrapancreatic lipid (IPL) accumulation and beta cell function, and whether this differs by ethnicity.

**Methods:**

Fifty-three BA (23 normal glucose tolerance, 11 impaired glucose tolerance and 19 type 2 diabetes) and 51 WE (23/13/15) men underwent a hyperglycaemic clamp and mixed-meal tolerance test to assess insulin secretion and beta cell function, a hyperinsulinaemic–euglycaemic clamp to measure insulin sensitivity and Dixon MRI to determine IPL. Associations between IPL and beta cell function were assessed using linear regression.

**Results:**

IPL was lower in BA compared with WE men (mean ± SD; 7.6 ± 2.6% vs 8.8 ± 3.7%, *p*=0.038), but after adjustment for waist circumference this ethnic difference no longer occurred (*p*=0.278). BA men with type 2 diabetes had lower total insulin secretion response to the mixed-meal (*p*=0.001) and hyperglycaemic clamp (*p*=0.002), but no ethnic differences were observed in the disposition index within glucose tolerance groups. IPL was inversely associated with beta cell function in the WE but not the BA men, but after adjustment for confounders these associations were not significant.

**Conclusions/interpretation:**

Ethnic differences were apparent as beta cell function was inversely associated with IPL in the WE, but not BA, men. However, in both ethnic groups, this relationship appears secondary to other factors, such as adiposity, in the pathogenesis of type 2 diabetes.

**Graphical Abstract:**

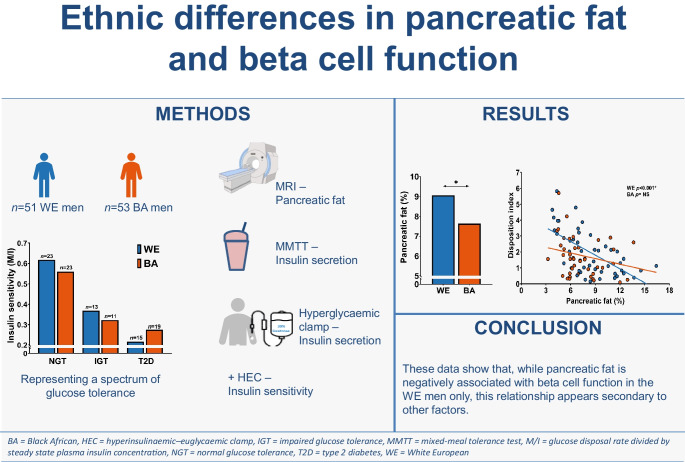

**Supplementary Information:**

The online version of this article (10.1007/s00125-025-06514-3) contains peer-reviewed but unedited supplementary material.


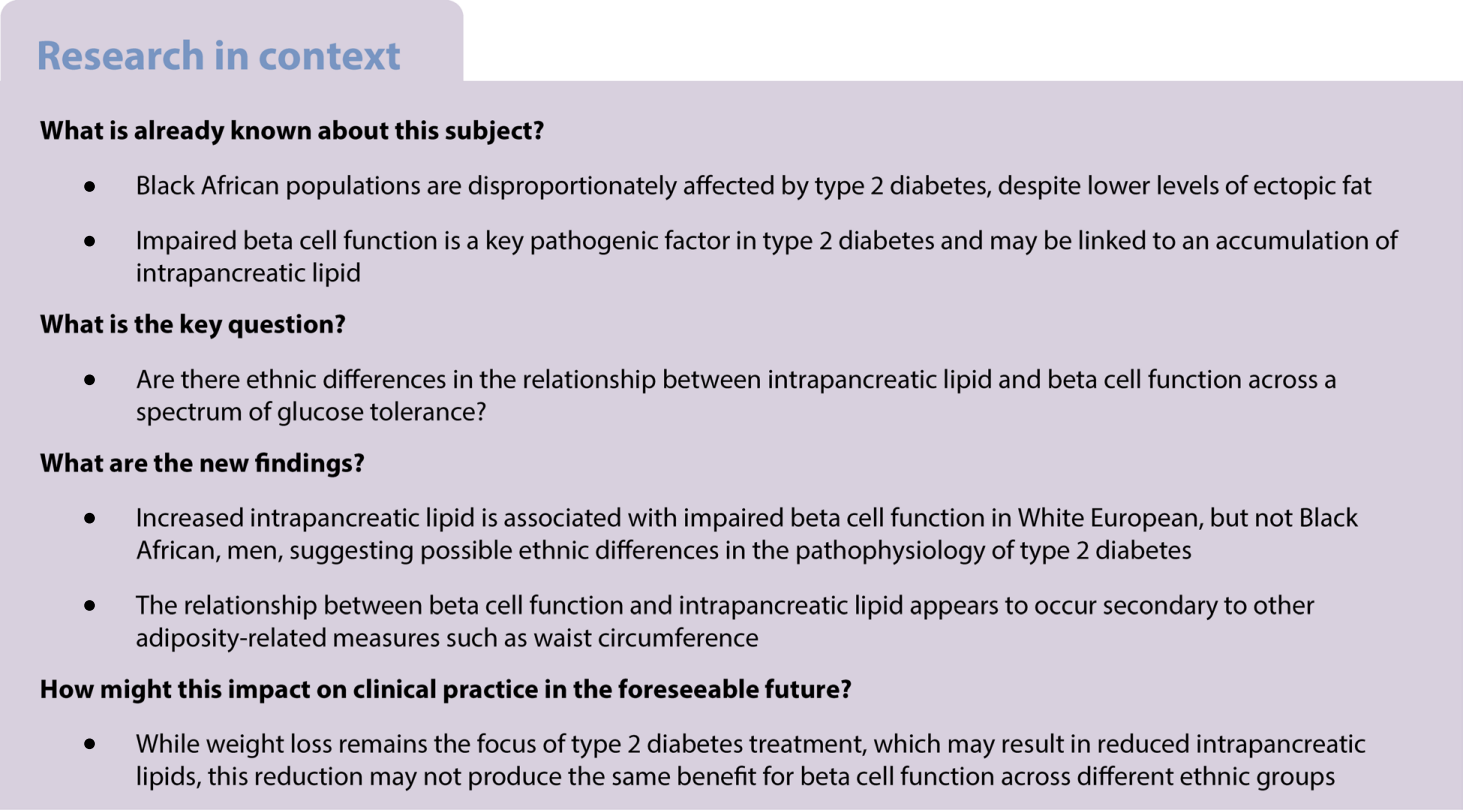


## Introduction

The role that intrapancreatic lipid (IPL) plays in the pathophysiology of type 2 diabetes is debated. While purported to drive impairments in beta cell function [1], inconsistencies in the methodological assessments of beta cell function, and failure to account for the underlying degree of insulin resistance, have generated inconsistent findings. People of Black African (BA) ancestry with, or at risk of, type 2 diabetes present with phenotypic distinctions including hyperinsulinaemia alongside low levels of visceral and hepatic fat compared with people of White European (WE) descent [2, 3], bringing into question the relevance of IPL in the development of hyperglycaemia among people of BA ancestry. The South London Diabetes and Ethnicity Phenotyping study (SOUL-DEEP) was set up to investigate ethnic differences in type 2 diabetes pathophysiology between BA and WE men across a spectrum of glucose tolerance [4]. We have previously observed lower IPL in BA compared with WE men with type 2 diabetes [5], despite similar IPL levels in men with normal glucose tolerance (NGT) [6]. Furthermore, we have found IPL to be associated with insulin secretion only in WE and not BA men [5–7]. We present here the full spectrum of glucose tolerance to assess the ethnic differences in the relationship between beta cell function and IPL, accounting for insulin sensitivity in our associative assessments, and including novel data from those with impaired glucose tolerance (IGT).

## Methods

Recruitment and data collection were conducted at King’s College Hospital and Guy’s Hospital, London, from April 2013 to May 2020 [4], and were approved by London Bridge National Research Ethics Committee (approval no. 12/LO/1859 and 15/LO1121). All participants provided written informed consent.

### Participants

Men (self-reported sex) aged 18–65 years of self-reported BA or WE ethnicity and with a BMI between 20 and 35 kg/m^2^ (inclusive) were eligible (full eligibility criteria reported previously [2] and further details in the electronic supplementary material [ESM] Methods). NGT and IGT were confirmed by oral glucose tolerance test; type 2 diabetes was confirmed in medical notes.

### Procedures

Full protocol details are available [4]. In brief, participants underwent a mixed-meal tolerance test (MMTT) and a hyperglycaemic clamp for assessment of insulin secretion and beta cell function, MRI for assessment of IPL and a two-step hyperinsulinaemic–euglycaemic clamp for assessment of insulin sensitivity. Details of the calculations of the outcome measures are provided in the ESM.

### Statistical analysis

The SOUL-DEEP studies were powered on a primary outcome of insulin secretory reserve (first-phase insulin response from the hyperglycaemic clamp). The type 2 diabetes study was powered on previously reported insulin secretion differences of 1 SD [8]; 20 per group would detect a difference of 1.0 SD, power 90%, significance level 5%. For the NGT and IGT studies we needed 23 per group to look at differences by ethnic group. However, we did not recruit sufficient IGT participants.

Participant characteristics were summarised by ethnicity and glycaemic group as mean (SD) for continuous variables and count (percentage) for categorical variables. Between-group differences were tested using independent sample *t* tests, ANCOVA (continuous variables) and χ^2^ tests (categorical variables). Associations between IPL (explanatory variable) and variables of beta cell function (outcomes) were explored with adjusted linear regression models fitted by ethnicity; confounders were selected using stepwise selection. The coefficient (95% CI) and *p* value for each model are presented. Missing data were not imputed, with analyses conducted only on complete cases. Statistical significance was assessed at the 5% level. Analyses were performed in Stata v18.0 (StataCorp, TX).

## Results

**Fig. 1:**
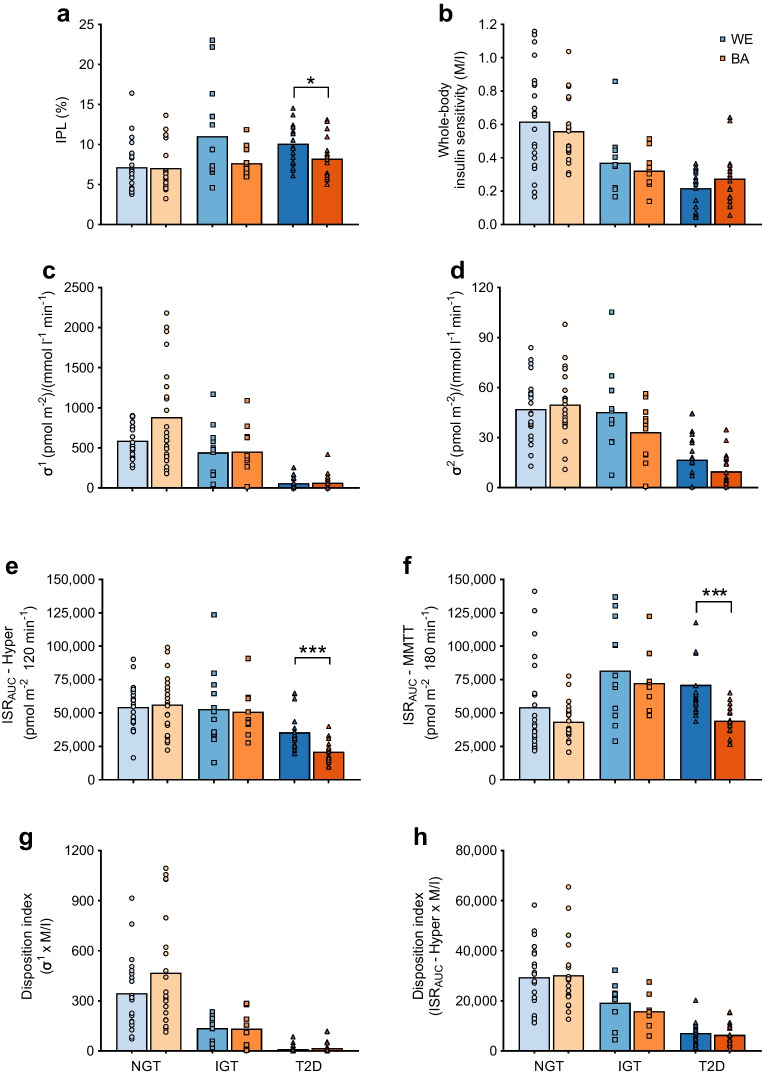
Metabolic characteristics of BA and WE men across a spectrum of glucose tolerance. IPL (**a**), whole-body insulin sensitivity (**b**), first- (σ^1^) and second- (σ^2^) phase insulin secretion from the hyperglycaemic clamp (**c** and **d**), total ISR from the hyperglycaemic clamp (**e**) and MMTT (**f**), and the disposition index calculated from both σ^1^ (**g**) and hyperglycaemic clamp ISR (**h**) are shown for BA and WE men with NGT, IGT and type 2 diabetes (T2D). The NGT group are represented by circles, the IGT by squares and the T2D by triangles. The WE group are represented in blue and the BA group in orange. Independent *t* tests were used to determine ethnic differences within each glucose tolerance group. *p<0.05, ***p<0.001. Data (**c**) were log-transformed before performing *t* tests. Insulin sensitivity data have been previously published [2]. Hyper, hyperglycaemic clamp; M/I, whole-body insulin sensitivity (glucose disposal rate divided by steady-state plasma insulin)

Participant characteristics are shown in ESM Table 1 and details of participant origin are provided in ESM Results. IPL, insulin secretion and beta cell function by ethnic group and glucose tolerance status are shown in Fig. 1 and ESM Table 2. Combining glucose tolerance groups demonstrated lower IPL in BA compared with WE men (7.6 ± 2.6% vs 8.8 ± 3.7%, *p*=0.038); however, when adjusting for waist circumference, IPL was not different between ethnicities (*p*=0.278). We have previously shown lower IPL in BA men with type 2 diabetes [6, 7].

The insulin secretion data from the NGT and type 2 diabetes groups have been published previously [6, 7], showing a lower insulin secretion rate (ISR) after both the oral (*p*=0.001) and intravenous (*p*=0.002) stimulus in the BA type 2 diabetes group (Fig. 1e, f). However, the disposition index, which accounts for insulin sensitivity in the assessment of beta cell function, showed no ethnic differences (Fig. 1g, h).

**Fig. 2:**
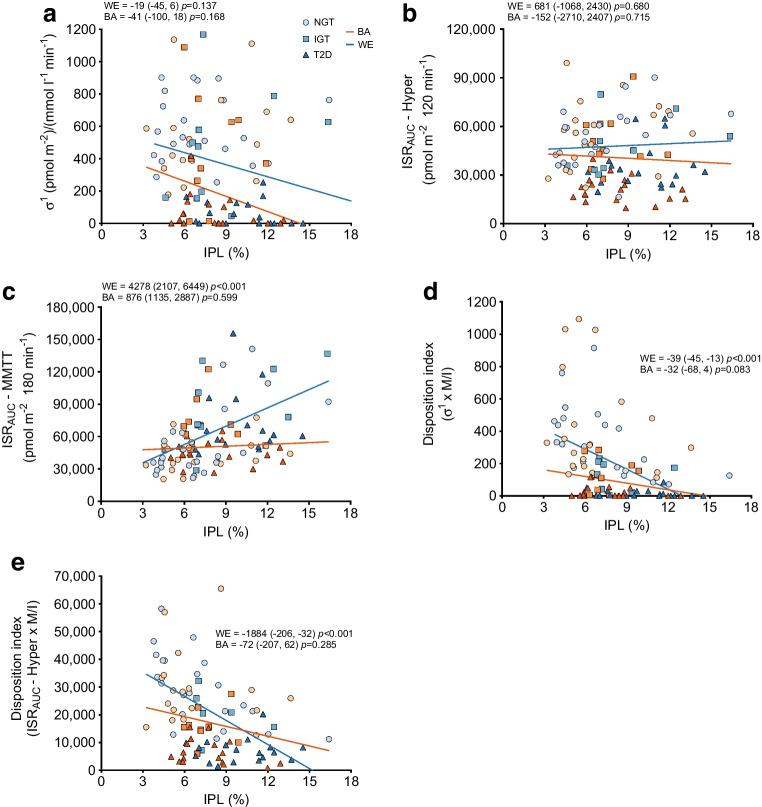
Ethnic differences in the relationship between IPL and beta cell function. The relationships of first-phase insulin secretion (σ^1^), total ISR from the hyperglycaemic clamp and MMTT with IPL are shown in (**a**), (**b**) and (**c**). The disposition indexes calculated from both σ^1^ and hyperglycaemic clamp ISR are shown in (**d**) and (**e**). In addition, unadjusted correlation coefficients and 95% CI values are included in each graph. WE men are represented in blue and BA men are represented in orange. The NGT, IGT and type 2 diabetes (T2D) are denoted by circles, squares and triangles, respectively. Hyper, hyperglycaemic clamp; M/I, whole-body insulin sensitivity (glucose disposal rate divided by steady-state plasma insulin)

Associations between IPL and the measures of insulin secretion and beta cell function are shown in Fig. 2 and ESM Table 3. First- and second-phase insulin secretion were not significantly associated with IPL in either the BA or the WE men (Fig. 2a). IPL was positively associated with total ISR from the MMTT and inversely associated with beta cell function (Fig. 2b, d, e), assessed by the disposition index from first-phase secretion (σ^1^) and total ISR during the hyperglycaemic clamp, in the WE men only. After adjustment for confounders (BMI, HbA_1c_, waist circumference, fasting plasma glucose, diastolic blood pressure, triacylglycerols and age) these relationships were no longer statistically significant (ESM Table 3).

## Discussion

In the present study we have examined ethnic differences in insulin secretion, beta cell function and IPL between BA and WE men across the full spectrum of glucose tolerance, and determined whether the relationship between IPL and beta cell function differs by ethnicity. We observed ethnic differences in IPL, whereby BA men with type 2 diabetes exhibited lower IPL compared with WE men; this occurred alongside greater total insulin secretion in response to both the mixed-meal and the hyperglycaemic clamp. Importantly, exploration of the associative relationship between IPL and beta cell function showed inverse associations in the WE but not the BA men.

Our analyses across the full spectrum of glucose tolerance show greater IPL in IGT and type 2 diabetes groups compared with NGT (when ethnic groups are combined), which aligns with the current understanding of the role of ectopic fat accumulation in type 2 diabetes pathophysiology, whereby a greater amount of fat accumulation is believed to cause organ dysfunction, leading to hyperglycaemia [9]. However, when split by ethnicity, this result was driven by the WE cohort, and the BA men had a much lesser increase in pancreatic fat across the glucose tolerance continuum (8% increase in IPL from NGT to IGT vs a 40% increase in WE men). In those with type 2 diabetes, we observed lower IPL in the BA compared with the WE ethnicity (8.1 ± 2.5% vs 10.0 ± 2.5%). This highlights how type 2 diabetes pathophysiology may be different in BA populations, and adds to our previous work that demonstrated lower ectopic fat in BA compared with WE men [2]. While there are currently few studies that have directly compared IPL levels in BA and WE populations, concurrent with an array of methodology used, our results align with the few that have been conducted. Lower IPL has been shown in Black adults compared with White and Hispanic adults with obesity [10], and also in Black women compared with White and Hispanic women [11].

A key finding from our study is that the inverse relationship observed between IPL and beta cell function exists only in the WE men, which suggests there may be ethnic differences in the role that IPL plays in the pathogenesis of type 2 diabetes, particularly regarding its impact on beta cell function. Our study is the first to assess this relationship across a spectrum of glucose tolerance in BA and WE men. Previous reports have been conflicting, which is likely due to the range of methodologies used to quantify insulin secretion/beta cell function. For example, a previous study assessing IPL and acute insulin response (assessed by intravenous glucose tolerance test), in a population without diabetes, showed a stronger relationship in the BA than the WE group [10], a study in women with varying degrees of glucose intolerance demonstrated a positive relationship between beta cell function (assessed by disposition index from intravenous glucose tolerance test) and IPL in BA women and an inverse relationship in WE women [11] and a third study in young obese adolescents demonstrated no relationship between the two variables within any ethnic group [12]. Taken together, this would suggest that there are ethnic differences in the relationship between beta cell function and IPL accumulation, but further research, including longitudinal studies, is required to fully unravel this relationship. Importantly, when we accounted for potential confounders, such as waist circumference, the observed associations were no longer significant. This suggests that IPL is unlikely to be causative, and that the decline in insulin secretion is likely related to another (adiposity distribution) factor, in both ethnic groups.

Our study has several areas of novelty and strength. Our dataset is the first to explore, using gold standard assessments, ethnic differences at three different glucose tolerance stages, in IPL, insulin secretion and beta cell function, between BA and WE men. Our use of a direct assessment of beta cell function is a superior methodological choice to other indirect measures [13]. Limitations of our present study include the insufficient number of participants recruited to the IGT group and the recruitment of men only. Although this was by design to obtain homogenous groups, ethnic differences by sex could not be investigated, and therefore a comparative study in women is needed.

In conclusion, ethnic differences were apparent due to the greater amount of IPL in the WE men, and the negative associations between beta cell function and IPL in WE men. However, these relationships appear to be secondary to other factors, such as overall and central adiposity, suggesting that IPL is not a primary determinant of beta cell function.

## Supplementary Information

Below is the link to the electronic supplementary material.

ESM (PDF 372 KB)

## Data Availability

The data that support the findings of this study are available from the corresponding author upon reasonable request.

## Abbreviations

BA: Black African
IGT: Impaired glucose tolerance
IPL: Intrapancreatic lipid
ISR: Insulin secretion rate
MMTT: Mixed-meal tolerance test
NGT: Normal glucose tolerance
SOUL-DEEP: South London Diabetes and Ethnicity Phenotyping study
WE: White European

## References

[1] Taylor R (2013) Banting memorial lecture 2012: reversing the twin cycles of type 2 diabetes. Diabet Med 30(3):267–275 23075228 10.1111/dme.12039 PMC3593165

[2] Whelehan G, Bello O, Hakim O et al (2024) Ethnic differences in the relationship between ectopic fat deposition and insulin sensitivity in Black African and White European men across a spectrum of glucose tolerance. Diabetes Obes Metab 26(11):5211–5221 39149769 10.1111/dom.15867

[3] Reed RM, Nevitt SJ, Kemp GJ, Cuthbertson DJ, Whyte MB, Goff LM (2022) Ectopic fat deposition in populations of black African ancestry: a systematic review and meta-analysis. Acta Diabetologica 59(2):171–187 34518896 10.1007/s00592-021-01797-5 PMC8841318

[4] Goff L, Amiel S, Umpleby M (2012) South London Diabetes and Ethnicity Phenotyping study protocol. University of Leicester. Available from: 10.25392/leicester.data.26213261.v1. Accessed 19 June 2025

[5] Hakim O, Bonadonna RC, Mohandas C et al (2019) Associations between pancreatic lipids and β-cell function in black African and white European men with type 2 diabetes. J Clin Endocrinol Metab 104(4):1201–1210 30407535 10.1210/jc.2018-01809

[6] Ladwa M, Bello O, Hakim O et al (2021) Ethnic differences in beta cell function occur independently of insulin sensitivity and pancreatic fat in black and white men. BMJ Open Diabetes Res Care 9(1):e002034 33762314 10.1136/bmjdrc-2020-002034 PMC7993168

[7] Mohandas C, Bonadonna R, Shojee-Moradie F et al (2018) Ethnic differences in insulin secretory function between black African and white European men with early type 2 diabetes. Diabetes Obes Metab 20(7):1678–1687 29516668 10.1111/dom.13283

[8] Hannon TS, Bacha F, Lin Y, Arslanian SA (2008) Hyperinsulinemia in African-American adolescents compared with their American white peers despite similar insulin sensitivity: a reflection of upregulated β-cell function? Diabetes Care 31(7):1445–1447 18417751 10.2337/dc08-0116 PMC2453672

[9] Lettner A, Roden M (2008) Ectopic fat and insulin resistance. Curr Diabetes Rep 8(3):185–191 10.1007/s11892-008-0032-z 18625114

[10] Szczepaniak LS, Victor RG, Mathur R et al (2012) Pancreatic steatosis and its relationship to β-cell dysfunction in humans: racial and ethnic variations. Diabetes Care 35(11):2377–2383 22968187 10.2337/dc12-0701 PMC3476895

[11] Lingvay I, Szczepaniak E, Szczepaniak L (2014) Ethnic diversity in beta-cell function susceptibility to pancreatic triglyceride levels: pilot investigation. J Diabetes Metab 5(348):2

[12] Lê K-A, Ventura EE, Fisher JQ et al (2011) Ethnic differences in pancreatic fat accumulation and its relationship with other fat depots and inflammatory markers. Diabetes Care 34(2):485–490 21270204 10.2337/dc10-0760 PMC3024373

[13] Muniyappa R, Lee S, Chen H, Quon MJ (2008) Current approaches for assessing insulin sensitivity and resistance in vivo: advantages, limitations, and appropriate usage. Am J Physiol Endocrinol Metab 294(1):E15–E26 17957034 10.1152/ajpendo.00645.2007

