# Supplementary material for: Ethnic differences in beta cell function and pancreatic fat in Black African and White European men across a spectrum of glucose tolerance
Source: Diabetologia. 2025 Aug 6;68(10):2290–5. doi: 10.1007/s00125-025-06514-3 (PMC12423149; doi:10.1007/s00125-025-06514-3)
Supplement: Supplementary file 1 — ESM (PDF 372 KB) [file 125_2025_6514_MOESM1_ESM.pdf]

Ethnic differences in beta-cell function and pancreatic fat in Black African and White European men across a spectrum of glucose tolerance

Gráinne Whelehan,[1,2], Oluwatoyosi Bello[3], Olah Hakim[4], Meera Ladwa[3], Danielle H. Bodicoat[5], A Margot Umpleby[6], Stephanie A. Amiel[3], Louise M. Goff,[1,2]

Electronic Supplementary Material

Methods

Participants

Participants were recruited from local primary care practices, newspaper advertisements, religious groups and leaflets. Men, aged 18-65 years, of self-reported Black (West) African (BA) and White European (WE) ethnicity, with a BMI between 20 and 35 kg/m^2 (inclusive) were eligible to take part. A 2-hour oral glucose tolerance test was used to confirm glycaemic status in those expected (on clinical grounds) to have normal and impaired glucose tolerance (1). Those with type 2 diabetes were required to have had a diagnosis within the last five years, to be treated by dietary intake and/or metformin with HbA1c of ≤64 mmol/mol. Participants were ineligible if treated with any medications known to affect study outcomes including thiazolidinedione, insulin, chronic oral steroids or beta-blockers, or if serum creatinine >150 µmol/L or serum alanine transaminase level >2.5-fold above the upper limit of the reference range. The aim of recruitment was to match BA and WE men within each glycaemic group for BMI and age.

Procedures

In the 24-hours before the study visits participants consumed a standardised diet including 50% energy from carbohydrate, with no more than 30% of daily carbohydrate consumed in the evening meal. The evening meal was consumed 10-hours before arrival at the Clinical Research Facility. Participants were asked to refrain from alcohol intake for 24-hours and physical activity 48-hours before the study visits. Participants attended four separate study visits for assessment of insulin secretion and beta-cell function, insulin sensitivity and intrapancreatic lipid.

Insulin secretion and β-cell function

Insulin secretion and beta-cell function were assessed in response to oral and intravenous stimulation using a mixed meal tolerance test and a hyperglycaemic clamp, respectively.

Mixed-meal tolerance test

The mixed-meal tolerance test was performed at the Metabolic Research Unit at King's College London. After a 10-hour overnight fast, participants rested in a seated position and an antecubital cannula was inserted into the non-dominant arm for blood sampling. Participants consumed a liquid milkshake (Ensure Plus; Abbott Nutrition, Berkshire, UK) containing 6 kcal/kg body weight; with carbohydrate (63%), protein (22%), and fat (15%). The mixed-meal tolerance test was consumed within five minutes. Blood samples were taken at −10, 0, 10, 20, 30, 40, 50, 60, 75, 90, 120, 150, and 180 minutes to assess plasma glucose, serum insulin and serum C-peptide.

Hyperglycaemic clamp

A 2-hour hyperglycaemic clamp was conducted to assess insulin secretion in response to intravenous glucose infusion (2). An antecubital cannula was inserted for intravenous infusion and a second cannula was inserted into the contralateral arm for blood sampling. Three fasting blood samples were collected (-20, -10 and 0 minutes). Thereafter, a 20% glucose infusion was administered to achieve a hyperglycaemic state (6.9 mmol/L above fasting plasma glucose concentration) for a period of two hours. Blood samples were obtained at 2, 4, 6, 8, 10, 15, 20, 30, 40, 50, 60, 75, 90, 105, and 120 minutes to assess plasma glucose, serum insulin and serum C-peptide.

Calculation and modelling of insulin secretion and beta-cell function

First- and second-phase insulin secretion, and total insulin secretion rate, were modelled from the hyperglycaemic clamp and the mixed-meal tolerance test: the glucose, insulin, and C-peptide curves from each test were modelled using methods previously described (3-5) and SAAM-II, version 1.2, software (SAAM Institute, Seattle, WA). The main outputs of the model were glucose sensitivity of first-phase insulin secretion (σ^1), expressed as the amount of insulin secreted in response to a rate of increase in plasma glucose concentration of 1 mmol/L between time zero and one minute of the study (pmol/m^2 body surface area)/(mmol/L/min) and glucose

sensitivity of second-phase insulin secretion (σ^2), expressed as the steady-state insulin secretion rate in response to a step increase in plasma glucose of 1 mmol/L above fasting concentration (pmol/min/m^2 body surface area)/(mmol/L).

We have used the disposition index as a measure of beta-cell function, whereby we have multiplied first-phase insulin secretion response and total insulin secretion rate from the hyperglycaemic clamp, by insulin sensitivity (measured in the hyperinsulinaemic-euglycaemic clamp [M/I; see calculation details below]).

Intrapancreatic lipid

Participants attended the Clinical Imaging Department at Guy's Hospital, King's College London, for the assessment of intrapancreatic lipid. A Dixon-based MRI sequence was used on a 1.5 Tesla Siemens scanner to acquire images to assess intrapancreatic lipids. With the participant lying supine, images were obtained from the neck to the knee (excluding the arms), with coils placed on the abdominal region. During acquisition of the abdominal images, on instruction by the radiographer, the participants undertook three 15-second breath holds to reduce the occurrence of motion artifacts. For each participant, 320 contiguous, 3-mm slice thickness, T1-weighted transverse spin-echo images (repetition time, 6.77 ms; echo time, 4.77 ms [in-phase], 2.39 ms [out-of-phase], flip angle, 10°) were obtained.

Intrapancreatic lipid was quantified by analysis of MRI scans using HOROS, version 1.1.7, software (available at: www.horosproject.org; accessed December 21, 2017) by locating one or more axial images with the largest area of the head, body, and tail of the pancreas and extracting the corresponding fat and water images. A circular region of interest of 1 cm^2 was drawn on the head, body, and tail of the pancreas on each of the fat and water images (6). Pancreatic lipid was calculated according to the below formula;

$$\% \text{ Pancreatic lipid} = [F/(F+W)] \times 100$$

where F is the pixel signal intensity of the fat image and; W is the pixel signal intensity of the water image. The pancreatic fat fraction was calculated in each region and the IPL^{MEAN} was calculated as the average of the head, body and tail regions. This is the measure of 'intrapancreatic lipid' used in the present analysis. Pancreatic lipid quantification was conducted by two independent investigators with a statistically significant correlation reported

(r = 0.62; P < 0.001) and an interobserver coefficient of variation of 14%. Paired samples t-test on the values obtained for IPL from both investigators revealed a P-value of 0.730.

Insulin sensitivity

Whole-body insulin sensitivity was measured during a two-step hyperinsulinaemic-euglycaemic clamp as previously described (7). Insulin sensitivity was calculated as glucose infusion rate 'M' divided by the change in serum insulin concentration from basal to Step 2 of the hyperinsulinaemic-euglycaemic clamp (M/I).

Biochemical analyses

Plasma glucose was measured using an automated glucose analyser (2300 STAT Glucose Analyzer; Yellow Spring Instruments, Yellow Springs, OH). Serum insulin was measured by immunoassay using chemiluminescent technology (ADVIA Centaur System; Siemens Health Care, Ltd., Camberley, UK). The inter-assay and intra-assay coefficients of variation ≤5.9% and 4.6%, respectively, for serum insulin. Serum C-peptide was measured by radioimmunoassay (Millipore Ltd., Hertfordshire, UK).

Results

Participants

The BA men originated from Nigeria (n=32), Ghana (n=14), Sierra Leone (n=2), Cote d'Ivoire (n=1) and Togo (n=1). All of the BA men in the IGT and T2D categories were first-generation West African migrants to the UK. 14 out of 23 of the BA men with NGT were second-generation West African, born in the UK.

ESM TABLE 1 Participant Characteristics

Characteristic	Normal Glucose Tolerance BA	Normal Glucose Tolerance WE	*P*-value[b]	Impaired Glucose Tolerance BA	Impaired Glucose Tolerance WE	*P*-value[b]	Type 2 Diabetes BA	Type 2 Diabetes WE	*P*-value[b]
n	23	23		11	13		19	15	
Age, years	30.7 (12.0)	35.9 (13.9)	0.183	45.7 (7.5)	54.5 (9.9)	**0.025**	54.1 (7.8)	55.5 (7.1)	0.602
Weight, kg	84.1 (14.6)	86.6 (16.6)	0.595	93.8 (9.8)	94.8 (17.0)	0.870	90.6 (9.2)	94.2 (11.6)	0.326
BMI, kg/m^2	26.7 (3.6)	26.5 (4.5)	0.873	30.0 (2.1)	29.8 (4.4)	0.917	29.5 (2.6)	30.1 (2.7)	0.510
Waist circumference, cm	87.5 (9.3)	93.8 (14.6)	0.088	100.8 (8.0)	105.7 (11.4)	0.249	103.7 (8.2)	107.5 (8.8)	0.194
Systolic BP, mmHg	123.2 (12.2)	121.9 (9.1)	0.687	134.1 (9.7)	130.1 (12.1)	0.387	137.3 (14.1)	131.8 (13.9)	0.262
Diastolic BP, mmHg	70.7 (11.5)	71.1 (8.2)	0.888	83.0 (7.2)	78.3 (6.6)	0.116	85.6 (7.4)	82.9 (10.1)	0.376
HbA1c, mmol/mol	37.0 (5.3)	35.9 (2.9)	0.372	43.2 (3.8)	39.0 (3.3)	**0.008**	49.9 (7.7)	48.6 (7.8)	0.631
HbA1c, %	5.5 (0.5)	5.4 (0.2)	0.373	6.1 (0.3)	5.7 (0.3)	**0.007**	6.7 (0.7)	6.6 (0.7)	0.650
Total cholesterol, mmol/L	4.3 (1.1)	4.8 (1.1)	0.126	4.4 (0.9)	5.0 (0.6)	0.081	4.1 (0.7)	4.3 (0.7)	0.470
LDL-cholesterol, mmol/L	2.7 (0.9)	3.0 (0.8)	0.191	2.7 (0.8)	3.1 (0.6)	0.271	2.3 (0.5)	2.3 (0.7)	0.794
HDL-cholesterol, mmol/L	1.3 (0.4)	1.3 (0.3)	0.753	1.2 (0.4)	1.3 (0.4)	0.670	1.2 (0.4)	1.2 (0.2)	0.557
Triglycerides, mmol/L	0.7 (0.3)	1.1 (0.6)	0.002	1.1 (0.4)	1.4 (0.5)	0.136	1.3 (0.7)	1.7 (0.7)	0.143
FPG, mmol/L	5.1 (0.4)	5.2 (0.4)	0.570	5.6 (0.5)	5.8 (0.7)	0.573	6.7 (1.0)	6.8 (1.4)	0.732
HOMA2-B	93.9 (26.9)	97.5 (37.3)	0.710	95.3 (26.5)	106.8 (49.7)	0.500	70.2 (7.8)	84.9 (44.3)	0.281
HOMA2-S	108.2 (43.7)	113.8 (61.2)	0.721	73.5 (23.7)	80.2 (12.7)	0.668	64.9 (30.6)	56.2 (28.9)	0.413
HOMA2-IR	1.1 (0.5)	1.2 (0.7)	0.608	1.5 (0.5)	1.9 (1.1)	0.319	1.9 (0.8)	2.4 (1.7)	0.220
Diabetes duration, years	-	-	-	-	-	-	2.8 (1.2)	2.9 (1.0)	0.815
Metformin use, count (%)							10 (53.3)	11 (73.7)	0.218

Data expressed as mean (standard deviation), unless otherwise stated. Abbreviations: BA, Black African; BMI, body mass index; BP, blood pressure; FPG, fasting plasma glucose; HDL-, high-density lipoprotein; LDL-, low-density lipoprotein; WE, White European. [a] Data were complete for all variables, i.e., there were no missing data. [b] Differences in means between ethnic groups were tested within each glucose tolerance group using the independent samples t-test for continuous variables and chi-squared test for categorical variables. P-values in bold represent statistical significance $P<0.05$.

ESM TABLE 2 Pancreatic fat and β-cell function markers by ethnicity and glycaemic status.

	Normal Glucose Tolerance					Impaired Glucose Tolerance					Type 2 Diabetes				
**Characteristic**	***n***	**BA**	***n***	**WE**	***P*[a]**	***n***	**BA**	***n***	**WE**	***P*[a]**	***n***	**BA**	***n***	**WE**	***P*[a]**
Pancreatic fat															
Pancreatic lipid, %	20	7.1 (3.0)	23	7.1 (3.1)	0.925	11	7.7 (1.9)	12	10.6 (6.2)	0.154	18	8.1 (2.5)	15	10.1 (2.5)	**0.029**
Insulin Secretion															
Hyperglycaemic Clamp:															
σ^1	23	882 (615)	22	588 (211)	0.190[b]	9	474 (349)	11	425 (335)	0.610[b]	19	67 (104)	15	68 (85)	0.429[b]
σ^2	23	50 (20)	22	47 (19)	0.649	10	35 (18)	11	46 (25)	0.251	19	10 (11)	15	16 (15)	0.202
ISR_{AUC}	23	56309 (21446)	22	54383 (16891)	0.742	10	51595 (18091)	11	52833 (30617)	0.913	19	21478 (7577)	15	33912 (13300)	**0.002**
Mixed-Meal Test:															
σ^1	21	3471 (2364)	23	3323 (1930)	0.819	8	3182 (1656)	8	2682 (1743)	0.509	17	1420 (1184)	15	1121(687)	0.394
σ^2	21	358 (294)	23	280 (244)	0.340	8	165 (89)	8	209 (97)	0.295	17	87 (83)	15	83 (41)	0.860
ISR_{AUC}	21	43389 (14307)	23	54276 (34036)	0.164	8	70188 (20254)	8	76728 (33459)	0.599	17	44861 (10152)	15	63796 (19317)	**0.001**
Disposition Index															
Hyperglycaemic Clamp:															
σ^1 x *Si*	21	468 (339)	22	345 (217)	0.163	7	147 (115)	9	136 (78)	0.832	18	25 (39)	15	17 (25)	0.511
σ^2 x *Si*	21	27 (15)	22	26 (14)	0.857	8	11 (7)	9	16 (9)	0.184	18	3 (4)	15	4 (5)	0.425
ISR_{AUC} x *Si*	20	30214 (13520)	22	29371 (13191)	0.839	8	17072 (5473)	9	19196 (8797)	0.565	18	6380 (4491)	15	8024 (4276)	0.293
Mixed-Meal Test:															
σ^1 x *Si*	20	1780 (1132)	23	1795 (1188)	0.968	8	922 (587)	8	943 (648)	0.946	17	507 (553)	15	302 (240)	0.193
σ^2 x *Si*	20	188 (185)	23	188 (262)	0.998	8	47 (15)	8	69 (56)	0.296	17	29 (30)	15	22 (16)	0.470
ISR_{AUC} x *Si*	21	22757 (6094)	23	25195 (5798)	0.181	8	23919 (6819)	8	26349 (9581)	0.568	17	12640 (7278)	15	14977 (5595)	0.322

Data expressed as mean (standard deviation), unless otherwise stated. Abbreviations: BA, Black African; WE, White European; σ1, first-phase insulin response ($(pmol·m^2)/(mmol·L·min^{-1})$); σ2, second-phase insulin response ($(pmol·m^2)/(mmol·L·min^{-1})$); Si, insulin sensitivity (M/I from hyperinsulinaemic-euglycaemic clamp; (mg/m^2 BSA min^{-1})/(pmol·L)); ISR_{AUC}, total insulin secretion rate ($pmol·m^2$ x120 min); [a] Differences in means between ethnic groups were tested within each glucose tolerance group using the independent samples t-test for continuous variables and chi-squared test for categorical variables. [b] Data were log-transformed before performing t-tests. P-values in bold represent statistical significance P<0.05.

ESM TABLE 3. Adjusted coefficient (95% CI) showing the association between pancreatic fat and beta-cell function markers[a]

**Beta-cell function marker**	**Total population (*n*=91)**			**BA (*n*=46)**			**WE (*n*=45)**		
	n	**Coefficient (95% CI)**	**P-value**	***n***	**Coefficient (95% CI)**	**P-value**	***n***	**Coefficient (95% CI)**	**P-value**
Hyperglycaemic Clamp:									
σ^1	90	-6.01 (-34.48, 22.47)	0.676	45	-27.81 (-80.83, 25.22)	0.295	45	3.47 (-27.60, 34.54)	0.822
σ^2	91	0.47 (-1.07, 2.00)	0.548	46	0.52 (-1.72, 2.77)	0.640	45	-0.41 (-2.89, 2.06)	0.738
σ^1 x *Si*	88	-4.25 (-20.11, 11.61)	0.595	43	-15.66 (-47.40, 16.09)	0.324	45	6.19 (-11.79, 24.17)	0.490
σ^2 x *Si*	89	0.13 (-0.77, 1.03)	0.779	44	0.00 (-1.62, 1.63)	0.996	45	0.27 (-0.99, 1.53)	0.663
Total ISR x *Si*	88	220.2 (-552.1, 992.5)	0.572	43	-131.7 (-1528.7, 1265.4)	0.850	45	607.6 (-426.0, 1641.1)	0.241
Mixed-Meal Test:									
σ^1 x *Si*	87	-23.06 (-101.74, 55.63)	0.561	42	-32.36 (-167.81, 103.08)	0.631	45	0.97 (-117.33, 119.26)	0.987
σ^2 x *Si*	87	-0.00 (-13.59, 13.58)	1.000	42	-6.45 (-25.20, 12.30)	0.490	45	6.48 (-16.66, 29.63)	0.574
Total ISR x *Si*	88	-64.7 (-652.5, 523.2)	0.827	43	-809.8 (-1756.7, 137.0)	0.091	45	399.9 (-428.2, 1228.0)	0.334

[a] *Estimated using linear regression models with pancreatic fat marker as the explanatory variable and beta-cell function marker as the outcome adjusted for BMI, HbA1c, waist circumference, fasting plasma glucose, diastolic blood pressure, triglycerides and age which were statistically significant confounders in stepwise selection. ; σ1, first-phase insulin response ((pmol·m^2)/(mmol·L·min^{-1})); σ2, second-phase insulin response (pmol·m^2)/(mmol·L·min^{-1}); Si, insulin sensitivity (M/I from hyperinsulinaemic-euglycaemic clamp; (mg/m^2 BSA min^{-1})/(pmol·L)); ISR_{AUC}, total insulin secretion rate (pmol·m^2 x120 min);*

1. American Diabetes A. 2. Classification and diagnosis of diabetes: standards of medical care in diabetes—2019. Diabetes care. 2019;42(Supplement 1):S13-S28.
2. DeFronzo RA, Tobin JD, Andres R. Glucose clamp technique: a method for quantifying insulin secretion and resistance. American Journal of Physiology-Endocrinology And Metabolism. 1979;237(3):E214.
3. Cobelli C, Toffolo GM, Man CD, et al. Assessment of β-cell function in humans, simultaneously with insulin sensitivity and hepatic extraction, from intravenous and oral glucose tests. American Journal of Physiology-Endocrinology and Metabolism. 2007;293(1):E1-E15.
4. Ladwa M, Oluwatoyosi B, Olah H, et al. Ethnic differences in beta cell function occur independently of insulin sensitivity and pancreatic fat in black and white men. BMJ Open Diabetes Research & Care. 2021;9(1):e002034.
5. Cali' AM, Bonadonna RC, Trombetta M, Weiss R, Caprio S. Metabolic abnormalities underlying the different prediabetic phenotypes in obese adolescents. The Journal of Clinical Endocrinology & Metabolism. 2008;93(5):1767-1773.
6. Al-Mrabeh A, Hollingsworth KG, Steven S, Tiniakos D, Taylor R. Quantification of intrapancreatic fat in type 2 diabetes by MRI. PloS one. 2017;12(4):e0174660.
7. Whelehan G, Bello O, Hakim O, et al. Ethnic differences in the relationship between ectopic fat deposition and insulin sensitivity in Black African and White European men across a spectrum of glucose tolerance. Diabetes, Obesity and Metabolism. 2024.
